# Evaluation of the acceptability of patient-reported outcome measures in women following pelvic floor procedures

**DOI:** 10.1007/s11136-022-03099-x

**Published:** 2022-02-03

**Authors:** Rasa Ruseckaite, Claire Bavor, Lucy Marsh, Joanne Dean, Oliver Daly, Dora Vasiliadis, Susannah Ahern

**Affiliations:** 1 Department of Epidemiology and Preventive Medicine, Monash University, Melbourne, VIC 3004 Australia; 2 Department of Obstetrics and Gynaecology, Western Health, Melbourne, VIC Australia; 3 Consumer Representative, Australian Pelvic Floor Procedure Registry, Melbourne, Australia

**Keywords:** Stress urinary incontinence, Registry, Acceptability, Quality of life

## Abstract

**Purpose:**

Patient-reported outcome measures (PROMs) are valuable tools in evaluating the outcomes of surgical treatment health-related quality of life (HRQoL) of women with stress urinary incontinence (SUI) and may be incorporated into related clinical quality registries. The aim of this study was to assess the feasibility and acceptability of incorporating PROMs into the Australian Pelvic Floor Procedure Registry (APFPR).

**Methods:**

Semi-structured qualitative interviews were conducted with women with SUI (*N* = 12) and their managing clinicians (*N* = 11) in Victoria, Australia. Interview topics covered content and face validity, appropriateness, and acceptability of three incontinence-specific, two pain, one anxiety and depression, one sexual function and one patient global impression of improvement instruments identified through the literature to determine their suitability and acceptability for the APFPR. We analysed interview data into topics using conventional content analysis.

**Results:**

Study participants agreed that PROMs were needed for the APFPR. Both participant groups suggested that some of the instruments were ambiguous, therefore only three instruments (one incontinence-specific, sexual function and patient global impression of improvement) will be included in the APFPR. Both clinicians and women agreed it would be appropriate to answer PROMs at baseline and then at 6- and 12-month postsurgically. Email, phone call and mail-out of the instruments were the preferred options for administration.

**Conclusion:**

Most women and clinicians supported the feasibility of incorporating PROMs in the APFPR. Participants believed the PROMs would demonstrate useful aggregate HRQoL data and have potential for use in individual care.

**Supplementary Information:**

The online version contains supplementary material available at 10.1007/s11136-022-03099-x.

## Background

In Australia up to 50% of women are affected by stress urinary incontinence, SUI [1], with a 20% lifetime risk of receiving a pelvic floor reconstructive procedure [2]. In addition, women with SUI have a significantly poorer health-related quality of life (HRQoL), yet procedures designed to relieve them of the condition may result in adverse events, such as chronic pain and vaginal mesh exposure, further diminishing their HRQoL [3, 4].

Patient-reported outcome measures (PROMs) are an important means of assessing HRQoL because they are reported directly by the patient, without interpretation by a clinician or anyone else [5]. A clinical registry is an efficient method of PROMs collection as it prospectively, routinely and systematically collects data from a large number of patients [6]. PROMs data from registries have been used to support observational studies which assess the association between patient demographics or disease burden and HRQoL [7, 8]. In Australia, several state and national registries currently use PROMs to evaluate impacts of treatment and complications on HRQoL whilst enabling health services and clinicians to benchmark their HRQoL outcomes against others [9–11].

The Australian Pelvic Floor Procedure Registry, APFPR [12] collects clinical and surgical information on patients undergoing pelvic floor procedures including any complications or adverse events related to the treatment from both surgeons and patients. The inclusion of PROMs in the APFPR is a critical activity providing the additional patient perspective of their condition prior to surgery as well as monitoring beyond the usual post-surgical follow-up time, adding to the information on both the effectiveness and safety of mesh-related procedures.

A number of PROMs have been developed for patients with pelvic floor disorders [13]. Careful consideration is required to choose the appropriate PROMs to include in a registry. In the context of a registry, limitations of using PROMs may be combined length and therefore the potential time burden for clinicians and patients in their ease of completion [6]. The aim of this study was to assess the feasibility of incorporating PROMs into the APFPR for patients and clinicians. This included the evaluation of the preferred mode and methods of administration and determining which instruments were the most suitable for patients and clinicians by assessing their relevance, clarity of wording, ease of use and clinical applicability.

## Methods

### Study design

We used a qualitative descriptive methodology to understand the personal perspectives and meaning of the study participants [14]. We conducted individual semi-structured telephone interviews to gather data.

### Participants

The study population consisted of adult women greater than 18 years of age with pelvic floor disorders who had previously undergone a procedure for SUI and clinicians treating SUI. The study was restricted to English-speaking participants. 10 to 15 women with SUI and 10–15 clinicians were expected to provide informative data and were sought purposively to ensure a mix of diversity in age, education and treatment experiences and outcomes. Data saturation was determined when no new information was generated from successive interviews.

### Recruitment

An advertisement with a brief outline of the study, a contact phone number and study email were developed. Female participants were recruited through social media via Facebook. Pelvic Floor Support groups on Facebook were contacted to request they post our advertisement on their page. The APFPR Steering Committee members and clinicians in our network were asked to advertise the study through their centres and networks.

We also recruited female participants through private clinician referrals by directly approaching the key clinicians in the APFPR network who perform SUI procedures across different demographic areas of Melbourne. The study advertisement was passed on to suitable patients with SUI by clinicians in their private clinics.

To invite Australian urologists, gynaecologists and urogynaecologists to participate in this study, an email was sent to the clinicians involved in the APFPR Steering Committee with a short advertisement of the study. Interested clinicians were encouraged to forward the invitation to their colleagues. The initial email was followed up by a reminder email 2 weeks later and, if required, a subsequent phone call.

Potential participants who expressed interest in the research were sent an explanatory statement describing the study and a copy of the instruments. It was not possible to establish how many participants saw the invitation to participate but decided not to volunteer. No participants dropped out of the research. Data saturation was determined when no new information was generated from successive interviews.

### Instruments

An extensive literature review was conducted to explore the pelvic floor procedure registries operating globally [15]. The review demonstrated that within these registries, a range of PROMs was used, but with little consensus on mode and methods of administration. Based on the findings of the literature review, the eight instruments described below were considered for this study.

The International Consultation on Incontinence Questionnaire Short Form, ICIQ-SF [16], the Incontinence Impact Questionnaire, IIQ-7 [17] and the Incontinence Quality of Life Questionnaire (I-QoL) have been validated for use in the measurement of urinary incontinence symptoms, daily activities, psychosocial impacts and social embarrassment in a variety of community and clinical populations both nationally and globally [18].

The Patient Global Impression of Improvement (PGI-I) instrument has been validated and widely used in both incontinence [19] and prolapse surgery [20]. The Pelvic Organ Prolapse/Urinary Incontinence Sexual Questionnaire-12 (PISQ-12) included in the study is a validated and reliable short form that evaluates sexual function in women with urinary incontinence and/or pelvic organ prolapse [21].

The Numerical Pain Rating Scale, NPRS [22] is a validated tool measuring pain intensity on an 11-point scale, taking less than one minute to complete. In addition, the Brief Pain Inventory, BPI [23] and the Hospital Anxiety and Depression Scale, HADS [24] were also included, with the expectation that if administered would only go to a small subset of women in the registry who have experienced a specific and defined level of pain and mental health complications.

### Data collection

Researchers developed semi-structured interview guides which used open-ended questions to assess participant views on the usefulness of the proposed instruments. An interview guide focused on the preferred mode to capture PROMs (i.e. self-administered or interviewer-administered), methods used to capture the information (such as a paper-and-pencil questionnaire or telephone- or computer-assisted technologies) and frequency of administration, content and face validity, appropriateness and acceptability of the eight instruments to determine whether they were suitable for inclusion in the APFPR. The interview guide has been included as online supplemental material. Interviews with either women or clinicians were conducted on the phone by two researchers experienced in conducting semi-structured interviews.

For trustworthiness, all interviewees were informed about the study purpose and confidentiality procedures. This was important for the interviewees to establish a setting were the participants felt that they could speak freely [25]. All interviews were conducted in a private environment on the phone. Follow-up questions and prompts were used to obtain rich data and all participants were offered the opportunity to review transcripts. The critical discussions held throughout the analysis facilitated both self-reflection and common discussion for distinguishing between participant meaning and research interpretation [26].

Prior to the interview, participants were instructed to review the instruments. Women with SUI were asked to report whether the items related to their everyday lives and health issues, and if not, what additional items could be added. Clinicians were further asked to consider the potential use of PROMs data in clinical consults. In addition, both groups of participants were asked about frequency, modes and methods of administration in the APFPR.

Women with SUI were offered a $20 gift voucher upon full completion of the interview in recognition of their contribution.

On average, interviews with women lasted 28 min (range 18–52) and with clinicians, 38 min (range 26–65). All interviews were audio-recorded, subject to the participants’ consent. The voice files were transcribed by paid transcription services. To ensure data quality, all transcripts were checked against the voice files by the interviewers.

### Data analysis

The consolidated criterion for reporting qualitative research (COREQ) 32‑item checklist was used for explicit reporting of the study methods [27]. Transcripts were analysed thematically: two members of the research team (RR, CB) first categorised responses into the themes embodied in the questions and then searched for any new themes raised by interviewees. One researcher began by coding phrases in the transcripts under key descriptive terms to create initial codes. These were combined into larger categories, which largely followed the interview guide; however, some novel categories emerged directly from the data. These categories were combined to form topics and subtopics, which have been defined and presented. To ensure coding rigour, a second researcher individually coded 10% of transcripts. Discrepancies between the two coders were resolved with discussion.

The process of analysing qualitative data involved coding and categorising the data from interview transcripts using NVivo software (Version 12, QSR, Australia). Transcripts were reviewed, and identifying quotes and words were grouped according to themes and sub-themes as they emerged from interviews with women and clinicians.

## Results

### Study participants

Twelve women with SUI with a mean age of 54.5 years (range 40–72) participated in the study (Table 1). Six of them had no complications. The average duration since their last procedure was 4.9 years ranging from 1 month to 10 years.

**Table 1:** Women with SUI characteristics

Code	Age (years)	Highest education	Time since the last procedure (years)	Complications (Yes/No)
001	67	Tertiary	2	Yes
003	49	High School	10	Yes
007	45	TAFE	14	Yes
008	63	High School	4	Yes
013	45	Tertiary	0.1	No
016	58	Tertiary	9	Yes
017	48	TAFE	10	Yes
020	40	Tertiary	1	No
021	68	Tertiary	1	No
028	45	Tertiary	2	No
029	54	Tertiary	0.5	No
031	72	TAFE	2	No

*SUI* Stress Urinary Incontinence, *TAFE* Technical and Further Education

Eleven experienced clinicians, consisting of 10 urologists (general gynaecologists or urogynaecologists or obstetrician surgeons and gynaecologists) and one nurse consultant participated in the study (Table 2).

**Table 2:** Clinician characteristics

Code	Years in practice	Number of SUI procedures per year	Specialty
005	7	30	Urogynaecologist/Obstetrician
010	25	500	Urological surgeon
011	26	80–90	Urogynaecologist
012	19	15–20	Urological surgeon
014	10	100	Urological surgeon
015	NA	NA	Clinical research nurse
018	19	10	Urogynaecologist/Obstetrician
019	16	50–60	Urogynaecologist
023	3	20	Urogynaecologist/Gynaecologist
024	30	50	Urogynaecologist
026	29	70	Urogynaecologist

### Evaluation of the instruments

#### Relevance of the ICIQ-UI instrument

All women with SUI agreed that this instrument was relevant, quick to complete, with good question choices and a good layout. The length of the instrument was also favoured by clinicians.

Many clinicians thought that the ICIQ-UI SF was relevant to the registry as it captured patient experiences following SUI procedures by assessing the nature of incontinence and its impact: “it informs the primary outcomes for the procedure” (C005). However, two clinicians noted that it did not assess whether patients were experiencing other mesh complications: “The trouble with most of these is they are good if you’re only looking at urinary incontinence” (C024). Some clinicians expressed concern that patients have trouble quantifying their incontinence and that their answers to the descriptive response options may not be accurate: “what one person could call it moderate another person could call it large or small” (C012). Similarly, some women said that a few questions were ambiguous making the instrument challenging to complete: “I found a lot of these forms quite difficult to complete. Mainly because I guess I didn’t feel that a lot of them actually fitted with my situation” (P001).

#### Comprehensiveness of the I-QoL

Despite varying opinions about the value of this instrument, four women with SUI referred to the instrument as relevant and straightforward: “it was very straightforward and you’ve got five different options” (P003). Some women felt that this instrument would not be relevant to their situation: “So it’s not really relevant for people who have had the mesh removed. Because, for instance, I mean, they talk about urine leakage but a lot of women afterwards have urge incontinence, rather than stress incontinence, after they’ve had their mesh removed” (P016).

All clinicians, except one, felt that the I-QoL was relevant to the registry, but too long: “I think it’s far too long, it’s far too detailed, there are very good questions in there” (C024). Whether clinicians believed its length was acceptable or not was often dependent on whether it was going to be answered by symptomatic or asymptomatic patients: “I think it’s quite long if somebody doesn’t have any problems. If they do, then the length of the survey may be warranted” (C005).

Some clinicians also felt that this instrument was repetitive: “I think there’s potentially a bit of crossover between the different questions….” (C005). In agreement with this, a few women with SUI did not understand some questions: “I’m trying to sort of work out was whether we’re only just talking about the continued incontinence or not after surgery? Or are we doing – is it to do also with the procedure and the problems that mesh give us after surgery?” (P001).

#### Irrelevance of the IIQ-7 due to outdated items

Almost all women with SUI and clinicians agreed that this instrument was very easy and quick to complete. However, some women thought that the response options were inconsistent: “They go the opposite way actually because this one goes ‘Extremely’ down to ‘Not at all’ and this one goes from ‘Not at all’ to ‘Greatly’” (P016).

One clinician believed the instrument was “completely outdated” (C012) as women were less likely to engage in the activities it asks about today than may they have when the instrument was developed. Similarly, there were comments that some questions assumed that the individual was participating in household and social activities: “because it makes some assumptions about people, like [entertaining], activities or you know, ability—you know, participation in social activities outside your home. Some people that’s going to be irrelevant to them” (C019).

#### Concerns about the use of pain instruments

Many women said that these instruments were irrelevant to them since they experienced no pain: “I didn’t really have any pain before. It was just incontinence, which to me wasn’t painful” (P031).

In terms of the NPRS, women were not clear what type of pain they were asked to describe: “Well actually I queried what pain is being measured, because my pelvic pain only? Or I’ve got other pain, like I’ve got the fibromyalgia pain. I’ve got car accident pain” (P001).

The clinicians believed that pain was an important outcome to assess. They believed the NPRS needed to specifically assess pain related to the procedure but many felt that it did not: “It captures their pain but it does not capture what it is due to or where the pain is or how the pain is related to particular time points” (C014). To resolve this, many suggested that “the question needs to be specific to the procedure” (C018) to ensure that participant responses were valid. Some clinicians noted that the instrument assessed acute pain and may not be suitable in a chronic pain population: “I think is not good because if they have pain six out of seven days or whatever, do you know what I mean, it’s not really a great pain question for chronic pain” (C012).

As for the BPI, women thought it was longer and “more specific”. However, the majority of clinicians were concerned that the BPI may capture pain unrelated to the procedure. They believed the questions needed to be more specific to ensure patients only responded in relation to pelvic pain: “What we should be only interested in is the pain related to our operation. So, you’ve got a whole body there and what happens when someone says ‘I’ve got pain’ and then they tick a shoulder?” (C018).

All participants expressed concern over the length of the BPI. Clinicians thought it would only be acceptable to administer to patients who indicated having pain on the NPRS: “I certainly think it’s relevant to those patients who are complaining of pain as a result of a procedure we’ve performed” (C026). They supported using the NPRS as leading questions to determine who should complete the BPI.

#### Support for the PGI-I

The majority of women with SUI thought that the PGI-I was relevant and easy to complete. However, they also believed that a definition was required for the procedure: “… are you talking pre-mesh, slight before, after mesh, after the mesh removal or now? I kind of had to look at it in four different contexts” (P001). Women suggested asking a leading question: “Maybe you need a question before that saying—because a lot of women haven’t had removal too” (P016).

The PGI-I was favoured by all clinicians for its short length and simplicity: “The beauty of this is a single question and you get really clear—patients really give good data on this question” (C010). They believed it was a “very important question” (C019) which succinctly captured the outcome of the surgery.

#### Mixed views towards the HADS

Many women with SUI agreed that this was a useful instrument; however, they provided mixed views. They thought it was important for their doctor to understand their emotional issues and feelings: “I did suffer problems from my incontinence, so I think it’s important to sort of understand how you’re feeling and how it’s affecting you” (P016). However, for a few women some of the questions seemed to be triggering: “It is a bit of a trigger. But when you go oh yes, I’m frightened about this and I don’t laugh much anymore and it just—oh, cripes, I really am badly depressed” (P003).

Many clinicians commented on whether it was appropriate for the registry to assess mental health outcomes. Some clinicians questioned whether the instrument captured mental health outcomes resulting from the surgery as they believed it was only “identifying those people who have anxiety or depression and comparing that before and after a surgical procedure” (C011).

#### Importance of the PISQ-12 instrument

The majority of the women with SUI believed that this instrument was relevant; however, they also “understood that some people would feel very uncomfortable answering the questions” (P029). Women suggested including an additional question, because not all of them were sexually active and, therefore, this instrument was not relevant to them: “it’s definitely important and it’s definitely relevant, but there has to be a question, are you sexually active?” (P021). Those completed this instrument agreed that it was very important: “I think 100% important this part. And I read through all these questions actually and I thought they were very relevant” (P020). Similarly, many clinicians suggested using a leading question: “I think a lot of this stuff is all irrelevant and I think that you need to actually ask the question right at the beginning, which is the first question that I ask any of my patients, starting with ‘are you still sexually active?’” (C018). Furthermore, if women were not sexually active, many clinicians highlighted the importance of asking why, as it may be due to the procedure: “There could be an important question on the top is, are you sexually active? Were you sexually active before the operation?” (C019).

The majority of clinicians felt sexual health outcomes were important to assess, but were concerned the PISQ-12 contained sensitive questions that could cause discomfort for some women. They were concerned that women may find it “quite confronting” (C011), “embarrassing” (C005) and “invasive” (C018). However, two clinicians believed that its importance outweighed these concerns: “So I think it can be sometimes a bit invasive and intrusive. Even though I think it’s really important to ask sexual questions, but if I had to choose between taking it all out and leaving it in, I’d leave it in” (C019).

### Frequency and timing for PROMs data collection in the APFPR

Many women with SUI agreed that PROMs should be administered frequently. The majority of women thought that every 3 to 6 months would suit them best. In general, women agreed that more frequent data collection was beneficial: “I think that annually would certainly be – but I mean if it was every three months, then you’ll most probably find that people would think it would be too long to be doing—and too much to be doing every three months. I would say that certainly annually would be—if not as minimum, six monthly, so half yearly, if not annually” (P007).

Two women agreed that baseline data were necessary and suggested PROMs be collected postsurgically and then annually: “I think maybe just post-surgery and then maybe the three months after and then a year later maybe” (P029).

Similarly, most clinicians also agreed that collecting PROMs at baseline, then at 3 to 4 months and annually would be appropriate: “I would do baseline and four months. I think by four months, most people have healed and they’re kind of back to their normal lives” (C019).

### Modes and methods for PROMs administration

All women preferred online PROMs administration; however, they also suggested having other options available, such as postal mail-out or phone for those women who were either not computer literate or did not have access to email: “I think you would probably have to give all women the option of that because there’s quite a lot of elderly women who are not computer literate. So, they would need the choice of whether they’d like to do it over the phone or by mail” (P013).

In general, women were open to the mode of data collection: “Well, I’m happy to talk on the phone, I’m also happy to answer another questionnaire and I could be happy to do that on an email. For me, I’m open to either format, but I think it’s important that you get someone professional that can answer some of your questions” (P021).

Clinicians thought that data collection should be made as simple as possible. If PROMs were to be captured in the clinic, paper-based methods would be the most common way to do this*:* “Electronic in the public sector tends not to be the common way of doing it. We’re all paper-based in the public sector here” (C023). They also believed that completing PROMs at the clinics would improve completeness of the data: “I think you’d get a better response rate, in terms of frequency response, if it’s done in a clinic. Because the doctor collects that data. But I think you may get a nicer response in the clinic” (C014).

## Discussion

PROMs are increasingly being implemented in clinical registries in Australia, to provide the patient’s perspective on the expectations and impact of treatment. Clinical registries play an increasingly important role as a stimulus for quality improvement by providing high-quality data and analyses that are respected by clinicians [28, 29]. PROMs in clinical registries are used for reporting and benchmarking purposes [29, 30].

The collection of PROMs by the APFPR is critical for providing additional information to support the safety monitoring of mesh-related adverse events. This is particularly important as the PROMs collected will provide baseline information about a participant’s condition prior to surgery as well as monitor them beyond the usual post-surgical follow-up time period [15].

We conducted qualitative interviews with women with SUI and experienced clinicians to determine the feasibility of incorporating PROMs into the APFPR. Eight instruments were reviewed and well accepted by both groups of participants. Both clinicians and women agreed that including PROMs in the registry would be beneficial. All participants agreed that the instruments need to be simple, easy to administer and short to complete.

Almost all participants suggested that the ICIQ-UI SF was the shortest, most clear and most relevant. There was high support for its inclusion in the registry. The ICIQ-UI SF is widely accepted and used in the other international registries, such as the British Society of Urogynaecology database [31], the Danish Urogynaecological Database [32] and the PFDR [33]. Additionally, the ICIQ-UI SF does not require training, it can be administered for free using the evaluation form and has been crossculturally validated in 63 different languages [34].

The PGI-I was the other well accepted and widely used instrument which was recommended for inclusion in the registry. This instrument is very simple, short and easy to use and is intuitively understandable to clinicians and women with SUI [19]. Based on participant feedback, a leading question will be included to specify the procedure the question refers to, as some women may have had more than one procedure in the past. The PGI-I has previously been used to assess HRQoL of women with SUI. For example, Nyström et al. [35] demonstrated that mean change in overall scores on ICIQ-UI SF correlated with the PGI-I as outcome measures. Larger reductions in the overall symptom score were associated with greater improvements (PGI-I) experienced after treatment.

Women with SUI and clinicians noted that PISQ-12 instrument [21] may make some women uncomfortable; however, none of the women in our study who were interviewed experienced this. This instrument has been successfully implemented in the PFDR, developed by the American Urogynecologic Society [33, 36]. This instrument was well accepted by our study participants; however, to address the concerns raised by participants, a leading question will be used since some women are not sexually active or prefer not to answer the questions in this instrument. Subsequently, this instrument was replaced by the Pelvic Organ Prolapse/Urinary Incontinence Sexual Questionnaire (PISQ-IR), a validated evaluation instrument which can be used clinically as well as in research for assessment of female sexual function in women with female pelvic floor disorders [37].

Two pain instruments (NPRS [22] and BPI [23]) were evaluated; however, the study participants did not think that either of these instruments were suitable for the registry. Women felt that the questions were too generic or were not applicable to them. Clinicians thought that pain was an important outcome in SUI and should be adequately assessed. Other pain instruments beyond the NPRS [22] and BPI [23] were later sourced and evaluated, but it was difficult to find one relevant and specific to the registry’s needs.

Women with SUI provided mixed responses towards the HADS [24]: the instrument was ambiguous, confusing, relevant only to a subset of patients, not useful, and relevant only for those with severe pain or mesh removal or its capacity to cause more anxiety. Based on these considerations, the team decided to exclude this instrument from the APFPR.

Participants agreed that PROMs should be collected at baseline (before the surgery or procedure) and then followed up at 6- and 12-month postsurgically. However, the date from the pre-surgical consultation to surgery can vary anywhere from 2 weeks up to 6 months and beyond, making it problematic to collect the initial pre-surgical PROMs. Email, phone call and mail-out of the instruments were the preferred options for administration. Most women participants believed electronic PROM administration would be efficient and easy to integrate with APFPR data; however, according to clinicians, completing PROMs in the clinic using paper-based methods may improve completeness of the data. Nevertheless, electronic PROMs administration costs less, improves data quality, results in similar or faster completion times and reduces administration times [38].

Limitations of this study included the small sample size of women with SUI and lack of culturally and linguistically diverse (CALD) participants. With this rapidly growing diverse population in Australia, understanding their views, needs and sensitivities is becoming very important in order to provide an appropriate treatment and assistance [39]. In addition, as the women did not have many complications, we were unable to determine the acceptability and feasibility of pain instruments due to lack of complications related to pain that may have resulted in particularly PROMs being deemed less relevant. Participants self-selected for the study based on an advertisement or email about PROMs. This may have captured non-representative sample of patients who are involved in research and more willing to review the instruments, and clinicians who are enthusiastic about PROMs. A strength of this study was to involve both women and clinicians this early in the process of PROMs integration in the APFPR. Therefore, this article provides an important view of their experiences and preferences using PROMs in the registry setting, which can support further implementation, feedback and utilisation of the data.

## Conclusion

Most women with SUI and clinicians supported the feasibility of incorporating PROMs into the APFPR. Participants believed the PROMs would demonstrate useful aggregate HRQoL data and have potential for use in individual care. They also emphasised that PROMs implementation must be supported by processes to feedback data to patients and clinicians, follow-up on red flags and opportunities to discuss potential areas of concern which arise following PROM completion. Most participants preferred electronic administration for easy integration with existing systems and the potential to support feedback. This study will be followed by evaluation study to identify which concepts each group found most relevant and important to measure, barriers and facilitators for PROMs data collection in the APFPR and evaluate the effect on shared decision-making, patient empowerment and clinical outcomes.

## Supplementary Information

Below is the link to the electronic supplementary material.

Supplementary file1 (DOCX 17 kb)

## Data Availability

Not applicable.

## Code availability

Not applicable.
