# Supplementary material for: Evaluation of the acceptability of patient-reported outcome measures in women following pelvic floor procedures
Source: Qual Life Res. 2022 Feb 3;31(7):2213–21. doi: 10.1007/s11136-022-03099-x (PMC9188490; doi:10.1007/s11136-022-03099-x)
Supplement: Supplementary file 1 — Supplementary file1 (DOCX 17 kb) [file 11136_2022_3099_MOESM1_ESM.docx]

# **Topic guide for patients**

Thank you for agreeing to be interviewed. Can I double check that you have read the Information and Consent Form? Do you have any questions before I begin?

As you are aware, this study is looking at patient and surgeon perspectives on including the ICIQ-UI SF, IIQ-7, I-QOL, the NPRS , PGI-I, BPI and the HADS questionnaires into the Australasian Pelvic Floor Procedures data registry. Have you had a chance to read the questionnaires and take some notes?

For the sake of accuracy, I’d like to record our conversation. Is that ok with you? I’ll put the phone on speaker. I want to assure you that everything we talk about will be confidential. If we write up our research, all your details will be de-identified.

1. First focusing on the ICIQ-UI SF, how did you find completing the questionnaire?
   1. Do you feel this questionnaire is relevant to the procedure and your experience following the procedure?
   2. Did you time how long it took you to complete the survey? What did you think of the length of the survey?
   3. Could you understand the questions? Did you need someone else’s help to complete it?
   4. Was there any ambiguity in the questions?
   5. Thinking about the response options, were these difficult to choose between? Could you suggest a better way of wording the responses?
   6. Were there any problems you encountered while completing the questionnaire?
2. Did you think the ICIQ-UI SF questions captured the most important and difficult parts of your experience with urinary incontinence?
   1. How precisely do these statements relate to your everyday life?
   2. Are there any questions you thought were irrelevant e.g. any symptoms you haven’t had trouble with at all?
   3. Are there any questions you would add?
3. Now focusing on the I-QOL; how did you find completing the questionnaire?
   1. Do you feel this questionnaire is relevant to the procedure and your experience following the procedure?
   2. Did you time how long it took you to complete the survey? What did you think of the length of the survey?
   3. Was there any ambiguity in the questions?
   4. Thinking about the response options, were these difficult to choose between? Could you suggest a better way of wording the responses?
   5. Were there any problems you encountered while completing the questionnaire?
4. Do you think the I-QOL questions captured the most important and difficult parts of your experience with incontinence?
   1. How precisely do these statements capture your experience? Are there any places you think the wording could be improved?
   2. Are there any questions you thought were irrelevant?
   3. Are there any questions you could add?
5. Now focusing on the IIQ-7;
   1. Do you feel this questionnaire is relevant to the procedure and your experience following the procedure?
   2. Did you time how long it took you to complete the survey? What did you think of the length of the survey?
   3. Could you understand the questions? Did you need someone else’s help to complete it?
   4. Was there any ambiguity in the questions?
   5. Thinking about the response options, were these difficult to choose between? Could you suggest a better way of wording the responses?
   6. Were there any problems you encountered while completing the questionnaire?
6. Do you think the IIQ-7 questions captured the most important and difficult parts of your experience with incontinence?
   1. How precisely do these statements capture your experience? Are there any places you think the wording could be improved?
   2. Are there any questions you thought were irrelevant?
   3. Are there any questions you could add?
7. Now let’s talk about the NPRS, how did you find completing the questionnaire?
   1. Do you feel this questionnaire is relevant to the procedure and your experience following the procedure?
   2. Did you time how long it took you to complete the question? What did you think of the length of the question?
   3. Could you understand the question? Did you need someone else’s help to complete it?
   4. Was there any ambiguity in the question?
   5. Thinking about the response options, were these difficult to choose between? Could you suggest a better way of wording the responses?
   6. Were there any problems or difficulties you encountered while completing the questionnaire?
   7. How relevant is pain to your everyday life?
8. Now let’s talk about the PGI-I, how did you find completing the questionnaire?
   1. Do you feel this questionnaire is relevant to the procedure and your experience following the procedure?
   2. Did you time how long it took you to complete the question? What did you think of the length of the question?
   3. Could you understand the question? Did you need someone else’s help to complete it?
   4. Was there any ambiguity in the question?
   5. Were there any problems or difficulties you encountered while completing the questionnaire?
   6. Do you think this question is relevant?
9. Now focusing on the BPI; how did you find completing the questionnaire?
   1. Do you feel this questionnaire is relevant to the procedure and your experience following the procedure?
   2. Did you time how long it took you to complete the survey? What did you think of the length of the survey?
   3. Was there any ambiguity in the questions?
   4. Thinking about the response options, were these difficult to choose between? Could you suggest a better way of wording the responses?
   5. Were there any problems you encountered while completing the questionnaire?
10. Do you think the BPI questions captured the most important and difficult parts of your experience with pain?
    1. How precisely do these statements capture your experience? Are there any places you think the wording could be improved?
    2. Are there any questions you thought were irrelevant?
    3. Are there any questions you could add?
11. Now focusing on the HADS; how did you find completing the questionnaire?
    1. Do you feel this questionnaire is relevant to the procedure and your experience following the procedure?
    2. Did you time how long it took you to complete the survey? What did you think of the length of the survey?
    3. Was there any ambiguity in the questions?
    4. Thinking about the response options, were these difficult to choose between? Could you suggest a better way of wording the responses?
    5. Were there any problems you encountered while completing the questionnaire?
12. Do you think the HADS questions captured the most important and difficult parts of your emotional recovery?
    1. How precisely do these statements capture your experience? Are there any places you think the wording could be improved?
    2. Are there any questions you thought were irrelevant?
    3. Are there any questions you could add?
13. Now that you’ve read these questionnaires, do you think the information they collect would be useful to include in the registry?
14. If these questionnaires are incorporated in the registry, how often would you be happy to fill out the questionnaire? E.g.) baseline, 6 weeks-3 months (post-operative appointment), and annually thereafter?
    1. What would be a barrier to filling out the questionnaire?
    2. Where would you want to fill out the survey; e.g. at home or at clinic?
    3. How would you want the survey to be administered; e.g. electronically, on paper?
15. A possible future use for the questionnaires is that they are made available to your doctors, who can talk to you about the results.
    1. Would you be happy with your doctor having access to these results?
    2. Do you think there are any possible advantages of disadvantages of being able to use these results in consultations with your doctor?

# **Topic-guide for clinicians**

Thank you for agreeing to be interviewed. Can I double check that you have read the Information and Consent Form? Do you have any questions before I begin?

As you are aware, this study is looking at patient and clinician perspectives on including the ICIQ-UI SF, the IIQ-7, I-QOL, NPRS, PGI-I, BPI and the HADS patient reported outcome measures into APFPR. Have you had a chance to read the PROMs and take some notes?

For the sake of accuracy, I’d like to record our conversation. Is that ok with you? I’ll put the phone on speaker. I want to assure you that everything we talk about will be confidential. If we write up our research, all your details will be de-identified.

1. First focusing on the ICIQ-UI SF;
   1. Do you feel this questionnaire is relevant to the procedure and your patients’ experience following the procedure?
   2. What did you think of the length of the survey?
   3. Could you understand the questions? Were there any ambiguity in the questions?
   4. Thinking about the response options, could you suggest a better way of wording the responses?
   5. Can you foresee any problems or difficulties for your patients while completing this questionnaire?
2. Do you think the ICIQ-UI SF questions captured the most important and difficult parts of your patients’ experiences with urinary incontinence?
   1. How precisely do these statements capture their experience? Are there any places you think the wording could be improved?
   2. Are there any questions you thought were irrelevant?
   3. Are there any questions you could add?
3. Now focusing on the I-QOL;
   1. Do you feel this questionnaire is relevant to the procedure and your patients’ experience following the procedure?
   2. What did you think of the length of the survey?
   3. Could you understand the questions? Were there any ambiguity in the questions?
   4. Thinking about the response options, could you suggest a better way of wording the responses?
   5. Can you foresee any problems or difficulties for your patients while completing this questionnaire?
4. Do you think the I-QOL questions captured the most important and difficult parts of your patients’ experiences with incontinence?
   1. How precisely do these statements capture their experience? Are there any places you think the wording could be improved?
   2. Are there any questions you thought were irrelevant?
   3. Are there any questions you could add?
5. Now focusing on the IIQ-7;
   1. Do you feel this questionnaire is relevant to the procedure and your patients’ experience following the procedure?
   2. What did you think of the length of the survey?
   3. Could you understand the questions? Were there any ambiguity in the questions?
   4. Thinking about the response options, could you suggest a better way of wording the responses?
   5. Can you foresee any problems or difficulties for your patients while completing this questionnaire?
6. Do you think the IIQ-7 questions captured the most important and difficult parts of your patients’ experiences with incontinence?
   1. How precisely do these statements capture their experience? Are there any places you think the wording could be improved?
   2. Are there any questions you thought were irrelevant?
   3. Are there any questions you could add?
7. Now we’ll talk about the NPRS;
   1. Do you feel this questionnaire is relevant to the procedure and your patients’ experience following the procedure?
   2. What did you think of the length of the question?
   3. Could you understand the question? Was there any ambiguity with the question?
   4. Thinking about the response option, could you suggest a better way of wording the response?
   5. Can you foresee any problems or difficulties for your patients while completing this questionnaire?
   6. How precisely does this question capture their experience with pain?
   7. Are there any places you think the wording could be improved?
   8. Is the question relevant?
8. Now we’ll talk about the PGI-I;
   1. Do you feel this questionnaire is relevant to the procedure and your patients’ experience following the procedure?
   2. What did you think of the length of the question?
   3. Could you understand the question? Was there any ambiguity with the question?
   4. Thinking about the response option, could you suggest a better way of wording the response?
   5. Can you foresee any problems or difficulties for your patients while completing this questionnaire?
   6. Are there any places you think the wording could be improved?
   7. Is the question relevant?
9. Now focusing on the BPI;
   1. Do you feel this questionnaire is relevant to the procedure and your patients’ experience following the procedure?
   2. What did you think of the length of the survey?
   3. Could you understand the questions? Were there any ambiguity in the questions?
   4. Thinking about the response options, could you suggest a better way of wording the responses?
   5. Can you foresee any problems or difficulties for your patients while completing this questionnaire?
10. Do you think the BPI questions captured the most important and difficult parts of your patients’ experiences with pain?
    1. How precisely do these statements capture their experience? Are there any places you think the wording could be improved?
    2. Are there any questions you thought were irrelevant?
    3. Are there any questions you could add?
11. Now focusing on the HADS;
    1. Do you feel this questionnaire is relevant to the procedure and your patients’ experience following the procedure?
    2. What did you think of the length of the survey?
    3. Could you understand the questions? Were there any ambiguity in the questions?
    4. Thinking about the response options, could you suggest a better way of wording the responses?
    5. Can you foresee any problems or difficulties for your patients while completing this questionnaire?
12. Do you think the HADS questions captured the most important and difficult parts of your patients’ experiences with their emotional recovery?
    1. How precisely do these statements capture their experience? Are there any places you think the wording could be improved?
    2. Are there any questions you thought were irrelevant?
    3. Are there any questions you could add?
13. Now that you’ve read these questionnaires, do you think the information they collect would be useful to include in the registry? Would the inclusion of all 7 questionnaires be beneficial?
14. If these questionnaires are incorporated in the registry, how often do you think the information should be collected to give useful data?

For example: Baseline, postop at 6 weeks-3 months, then annually thereafter?

- 1. Would you be happy to collect this data at baseline?
  2. Who do you think is best to administer the question – the Patient, the Clinician, or Other” and why?
  3. What would be optimal timing for baseline PROM administration?
  4. What are possible barriers you can foresee to patients filling out the questionnaire?
  5. How often do you think patients will be happy to fill out the questionnaire?
  6. Where would you want patients to fill out the surveys, e.g. at home or at clinic?
  7. How would you want the survey to be administered, e.g. electronically or on paper?
  8. What are the advantages and disadvantages for patients and clinical staff of each method?

1. A possible future use for the questionnaires is that they are made available to doctors, who can use the results to track patient quality of life and discuss the results in a consultation.
   1. Do you believe incorporating PROM data could enhance your practice?
   2. Can you expect any negative consequences from this?
   3. Can you foresee any barriers to implementing this?
